# Translational microbiomes in agriculture: microbial communities as tools to effect host and system health for improved crop production

**DOI:** 10.1128/aem.01704-25

**Published:** 2026-01-08

**Authors:** Alex D. Vompe, Mozhde Hamidizade, Melanie Medina López, Eoin O'Connor, Livleen Kaur, Kevin L. Hockett, Carolee T. Bull

**Affiliations:** 1 Department of Plant Pathology and Environmental Microbiology, The Pennsylvania State University 8082 , University Park, Pennsylvania, USA; 2 One Health Microbiome Center, The Pennsylvania State University 8082, University Park, Pennsylvania, USA; 3 Department of Food Science, The Pennsylvania State University 8082, University Park, Pennsylvania, USA; University of Georgia Center for Food Safety, Griffin, Georgia, USA

**Keywords:** translational microbiomes, One Health, SynCom, passaging, crop health, crop management, resilience, seed microbiomes

## Abstract

The boom of microbiome research in agriculture over the past several decades allows scientists, growers, policymakers, and businesses to collaborate on a unique opportunity—deploying microbiomes and microbiome attributes for the improvement of crop production. The idea of translational microbiomes is well established in the medical field; however, this framework is relatively new to agriculture. In this review, we discuss a series of methodologies grounded in microbiome science to enhance crop health. These include diagnostic approaches (pathogen and toxin detection and the monitoring of stress-related community ecology patterns) and intervention strategies (synthetic communities, microbiome-aware crop management practices, passaging microbiomes, and exploiting the vertical and lateral transmission of microbiomes to seeds). Developing and implementing these approaches remain challenging due, in part, to a shortage of long-term *in situ* studies demonstrating the robustness and effectiveness of translational microbiome efforts against the background of heterogeneity and ecological complexity of agricultural systems. Moreover, the cost and availability of ‘omics methods central to microbiome analysis, disparate standards for microbiome product development, and limited longstanding relationships with stakeholders have slowed down the application of microbiome-based solutions. However, the increasing cost-effectiveness of microbiome approaches in crop management makes translational microbiomes likely assets in the movement toward precision agriculture. This “personalized treatment” for plants holds promise for improved food security and environmental sustainability, by reducing commonplace synthetic amendments and promoting native microbial biodiversity.

## INTRODUCTION

**Fig 1:**
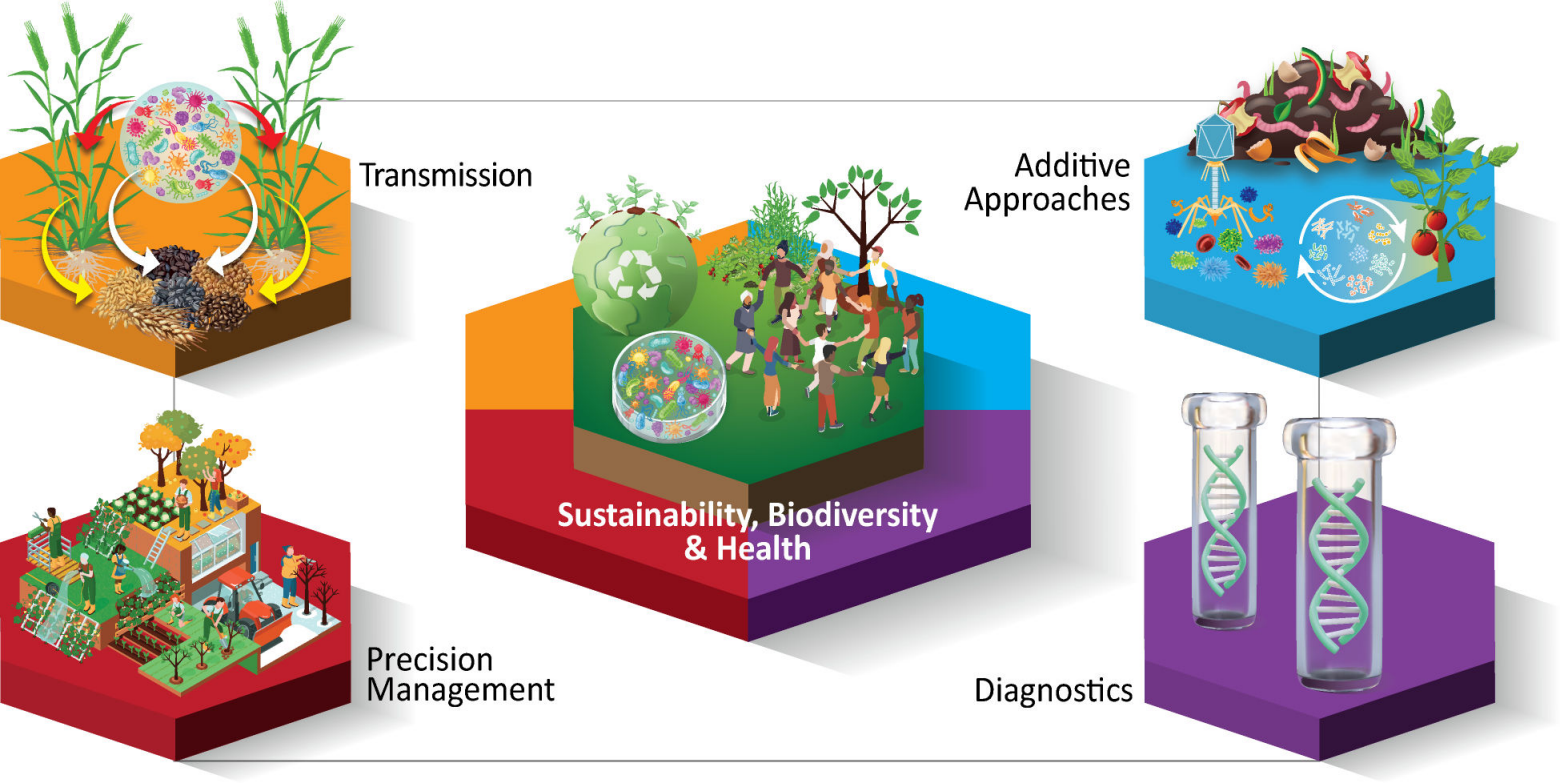
Translational microbiomes in crop production contribute to the One Health approach by leveraging diagnostics, precision management, microbiome transmission mechanisms, and additive approaches to enhance sustainability, biodiversity, and health.

Since 2015, the term **Translational Microbiome** (Table 1 provides definitions of all bold terms) has been applied almost exclusively to refer to the use of knowledge of human microbiomes for clinical applications to improve patient health. Solutions for clinical problems are emerging from over 10 years of presentations at annual conferences and publications integrating microbiome sciences and medical research. While the term has not been applied to agricultural microbiomes, we apply the translational microbiome framework to describe progress made toward utilizing microbiome knowledge to develop applications for crop health and production. Using this approach, we hope to enable the discovery of unifying patterns across hosts and environments for agricultural advancement (Fig. 1).

**TABLE 1:** Glossary of key terms

Term	Definition
‘Omics	Molecular and bioinformatic approaches to survey the comprehensive diversity of biomolecules of interest in samples from complex environments
Alpha diversity	A measure of the taxonomic diversity of a single community
Beta diversity	An enumeration of differences between the members of two or more communities
Biochar	A black solid material composed of carbon generated from the pyrolysis of biomass
Biocontrol	The application of living organisms to control the proliferation of a pest or disease
Competitive exclusion	A population of microbes outcompeting another population of microbes for space and resources to elimination from the community
Dysbiosis	A microbiome state that associates with host stress, disease, and decline
Endophytically	Within plant tissues
Epiphytically	On the plant surface
Eubiosis	A microbiome state that associates with host health and resilience
Fitness	Ability to reproduce successfully
Functional diversity	A rich functional repertoire, often associated with taxonomically diverse microbiomes
Functional redundancy	A community state in which the taxonomic diversity is greater than the functional diversity (i.e., multiple different organisms fulfill the same functions)
Lytic life cycle	A viral life cycle that involves the rupture and death of the host to release newly formed viral particles
Mature microbiomes	Established microbiomes of mature hosts
Metagenomic	Genomic data from all organisms and viruses in an environment
Pathobiomes	Microbial communities associated with pathology
Phytobiomes	Plants and their associated communities of organisms and interactions with their environment
Prebiotics	A type of soil biostimulant that acts as a selective substrate for beneficial microbial communities in the soil or phytobiome, enhancing microbial function that results in improved plant health
Resilience	The ability to recover following a disturbance
Richness	The number of different types of microorganisms in a microenvironment
Seed microbiome	The community of the microorganisms including bacteria, fungi, viruses, and archaea associated with seeds
Soil amendment	A product that aims to improve the physical, chemical, or biological properties of soil that results in an improved plant health outcome
Soil biostimulant	A substance or microorganism that is added to soil in an effort to improve traits such as plant vigor, nutrient efficiency, and/or disease resilience, regardless of its nutrient profile
SynComs	Synthetic communities: defined and engineered assemblages of microorganisms to study microbial interactions
Terroir	The influence of the environment and microbes on crop flavor, aroma, and quality
Translational microbiome	The use of microbiomes to improve the health and resilience of a host of interest

Beyond human microbiomes, **phytobiomes** are an integral part of One Health approaches (1), which makes them compelling targets for the application of a translational microbiome framework. Phytobiomes can impact human and environmental health through a myriad of interactions, and phytobiome interventions can change their outcomes. The most obvious impacts are on food security. Ensuring food security presents numerous challenges, including the need to increase crop yields and prevent plant diseases, to meet the nutritional demands of a growing population. Climate change further complicates these issues by disrupting environmental conditions, leading to more frequent extreme weather events, and influencing the distribution patterns of plant diseases (2, 3). Extensive research has demonstrated the effectiveness of plant growth-promoting microorganisms in mitigating the effects of environmental stresses and plant disease on crop performance (4, 5).

Food safety is also an important challenge in agricultural systems, and the use of microbes to mitigate the risk of foodborne illness shows great potential (6, 7). Compounding this issue, antimicrobial resistance transferred to foodborne pathogens from commensal organism carriers is of particular concern (8). Antimicrobial resistance genes found on plasmids in phyllosphere, rhizosphere, and endophytic organisms (either commensal or plant pathogenic) may be considered reservoirs of antimicrobial resistance (9). Management of phytobiomes or environmental microbiomes has implications for the transfer of resistance genes that may increase or mitigate the emergence of antimicrobial resistance in human pathogens (10).

Translational microbiomes promise to reduce chemical pesticide use, thus lowering the exposure of farm workers and others to chemical pesticides, minimizing potential impacts to their health (11). Microbial pesticides are also employed to manage pest populations that are resistant to chemical pesticides (12). Beyond single microbe pesticides, consortia are being developed to degrade chemical pesticide contamination (13). Microbiome manipulation through crop management practices discussed below may also increase profitability.

While microbiome manipulations and microbial inoculants present promising solutions to many problems in sustainable agriculture and food safety, their practical application has proven challenging, reflecting the complexity of agricultural systems, which vary widely, from tightly controlled-environment agriculture facilities to open-field production, where environmental factors play a major role. This variability makes it difficult to implement generalized microbiome-based solutions. Nevertheless, in this review, we highlight innovative translational microbiome approaches that may address food security and safety challenges.

## MICROBIOMES AS SYSTEM HEALTH DIAGNOSTICS

**Fig 2:**
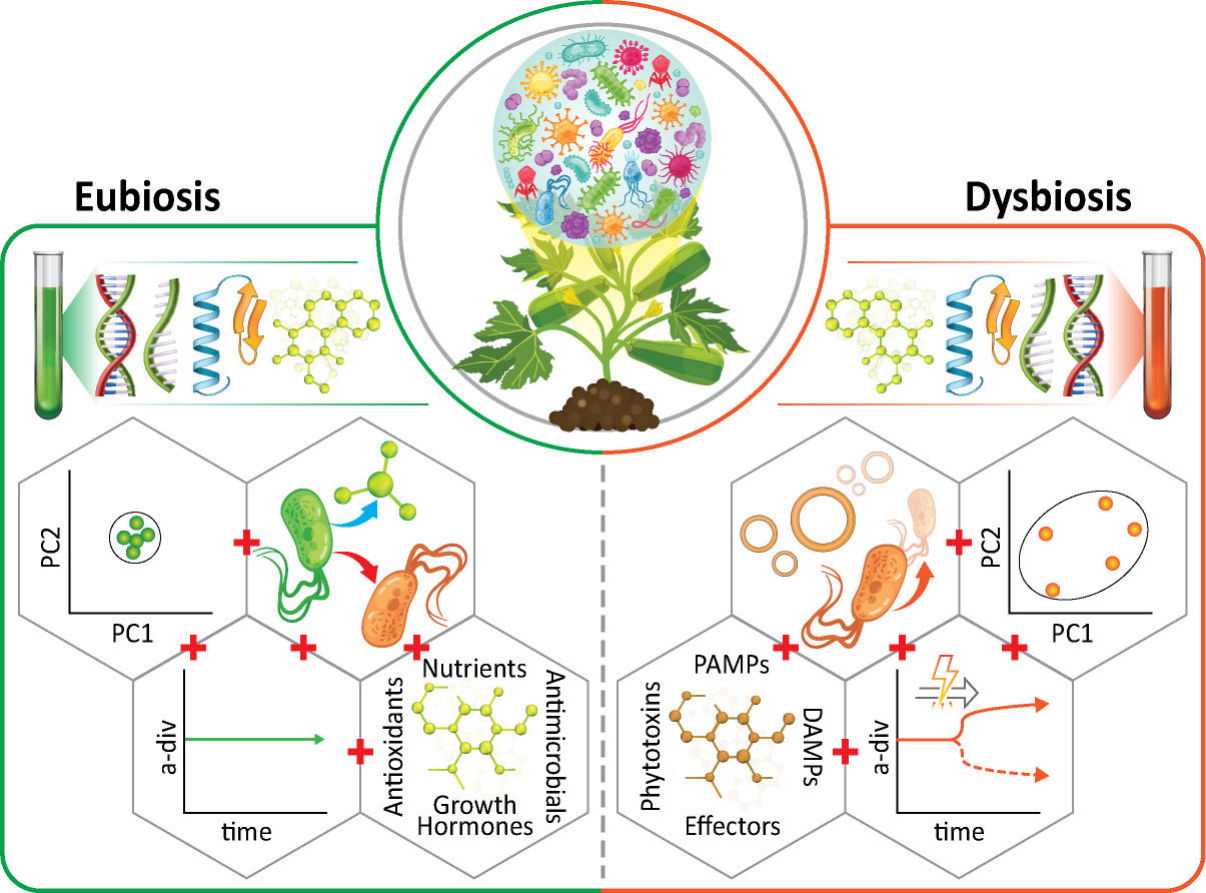
Microbiomes as diagnostics for crop production. ‘Omics analyses of macromolecules (DNA, RNA, proteins, metabolites, and others) can help diagnose crop microbiomes or substrates as eubiotic or dysbiotic. Key microbiome metrics shown here should be considered together when making microbiome-informed assessments of crop system health. Left panel (Eubiosis, left to right, top to bottom): low beta dispersion for crop systems adhering to AKP, pathogen suppression, and beneficial metabolite production as determined from network analysis, stable alpha diversity, and the detection of nutrients, antimicrobials, growth hormones, and antioxidants. Right panel (Dysbiosis, right to left, top to bottom): high beta dispersion for crop systems adhering to AKP, metagenomic analysis pathogen taxonomy and evolution, abrupt change in alpha diversity, detection of PAMPs, DAMPs, effectors, and phytotoxins.

While microbiome manipulations in crop production can have important translational consequences (as discussed in the sections below), the native microbiomes themselves can sometimes serve as valuable diagnostics of system or host health (Fig. 2) (14–16). There is evidence of patterns of **eubiosis** and **dysbiosis** across animal and plant microbiomes. The Anna Karenina Principle (AKP) emerges as a pattern of dysbiosis in many plant and animal microbiomes (1, 17–19). The AKP comes from the first lines of Leo Tolstoy’s novel “Anna Karenina,” “all happy families are alike, each unhappy family is unhappy in its own way,” asserting, by analogy, that it is common for healthy microbiomes to be more deterministic than ones associated with stress, disease, or lower **fitness** (19, 20). As such, an increase in the **beta diversity** metric of dispersion (microbiome stochasticity) is frequently a proxy for dysbiosis. Multiple studies have documented this phenomenon in crop and plant stress, including broad patterns of agricultural soil heat shocking (21), bacterial-wilt-associated tomato rhizosphere soil (22), *Fusarium* wilt diseased pepper plants (23), and others (24–26). While the AKP widely applies to animals and plants, there are notable exceptions to the rule. For example, some conditions, such as soybean and rice root nematode infections, actually associate with more homogeneous microbiomes than their healthy counterparts, a pattern called “anti-AKP” (17, 27, 28). Interestingly, these patterns may sometimes depend on the pathogen load. For example, *Erysiphe alphitoides* infection in oak (*Quercus robur* L.) follows AKP patterns at low pathogen loads; however, dysbiotic microbiomes in hosts with higher pathogen loads follow anti-AKP, where the pathogen makes microbiomes more deterministic (29). Such patterns may occasionally be attributable to pathogen dominance in the microbiome; however, in the examples above, the pathogens were external to the communities experiencing shifts in beta diversity (shifts were observed in fungal communities under either nematode challenge [28] or fungal pathogen challenge, in which case the pathogen was not included in beta diversity analyses [29]).

Other markers of microbiome health and **resilience** in crop systems include identifiable genes associated with host dysbiosis, as well as changes in **alpha diversity**, patterns in taxonomic relative abundance, and the presence of certain transcription factors. First, microbiome dysbiosis can be associated with a high abundance or expression of microbial genes that trigger host immune responses. Examples include pathogen-associated molecular patterns (PAMPS), damage-associated molecular patterns (DAMPS), effector genes, plant cell wall degrading enzyme genes, phytotoxins, quorum sensing genes, and secretion systems (30). Next, significant changes in alpha diversity (sometimes increases, sometimes decreases) of **mature microbiomes** are frequently indicators of dysbiosis in plant systems. This variable link between taxonomic diversity and health is likely attributable to either **functional diversity** or **functional redundancy** (31, 32). Some examples include drought significantly affecting rice mycobiome alpha diversity and impaired immunity significantly reducing *Arabidopsis* microbiome alpha diversity (33, 34). Microbiome taxonomic composition is also important to consider, as some taxa are generally seen as pathogens or commensals across plant hosts or can be defined as such in curated databases (35). The **metagenomic** quantification of microbiomes is becoming an indispensable tool for detecting rapidly evolving pathogens or diverse **pathobiomes** with high precision (36). Finally, there are conserved host transcriptome effects on microbial community assembly. For example, the root transcription factor MYB72 induces systemic resistance pathways, which select for beneficial taxa, while inhibiting pathogens and opportunists (14, 37). It is also important to consider that when applying microbiomes as diagnostic tools, it is often unclear whether the microbiome state causes or results from the host phenotype or system change. Thus, experiments clarifying the nature of the association are critical.

The expensive, technically, and computationally demanding ‘omics methodologies required to quantify the above microbiome metrics currently hinder the integration of microbiomes as system health diagnostics in agriculture. However, there are several ways to lower these barriers, including pooling samples across a region of interest, developing and providing standardized protocols, and partnering with diagnostic labs specializing in ‘omics techniques (35, 36, 38). Some companies are already providing microbiome data to growers.

## INTERVENTION STRATEGIES

**Fig 3:**
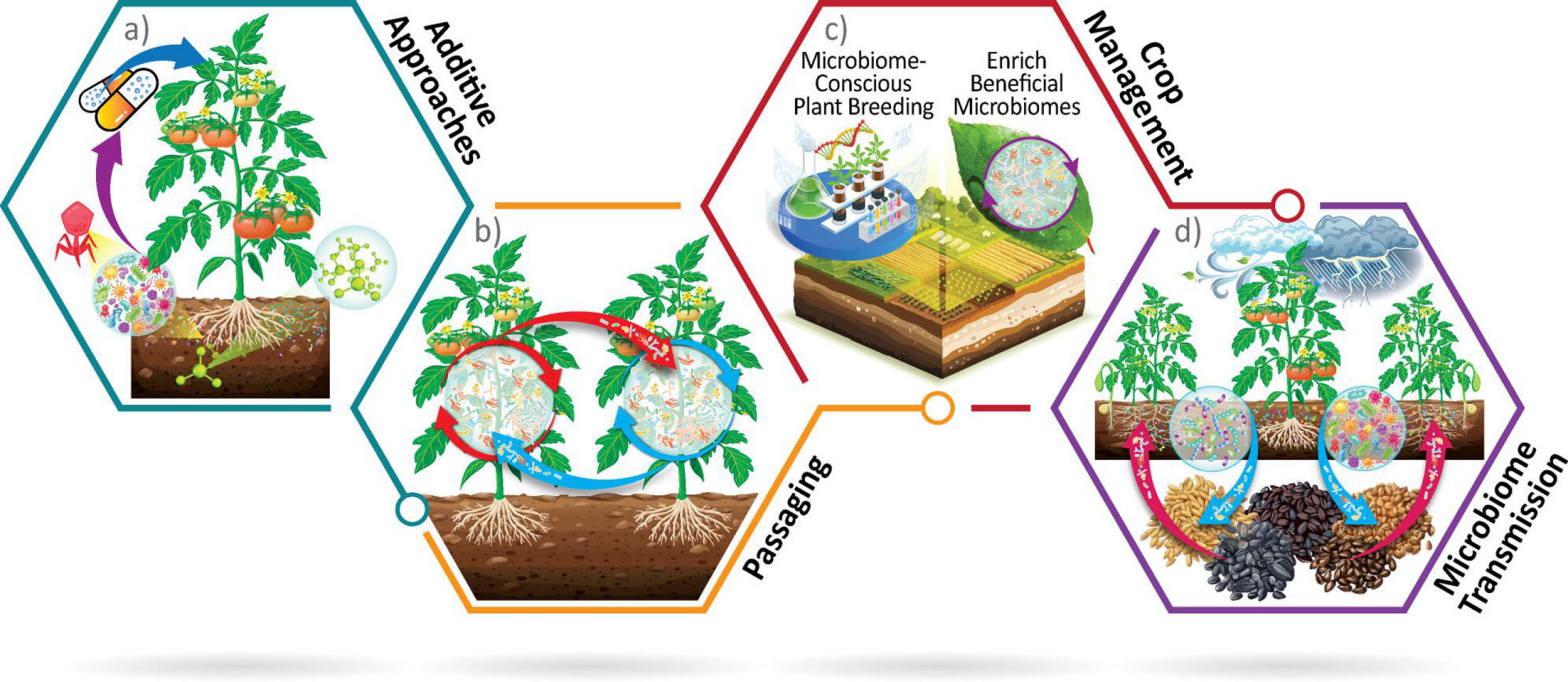
Translational microbiome intervention strategies. Additive approaches, including prebiotics, probiotics, and SynComs can enrich or employ native microbial flora to improve host health and resilience (**a**); passaging approaches are selective *in vivo* processes that suppress pathogens in the phyllosphere and rhizosphere (**b**); microbiome-aware crop management, including microbiome-aware plant breeding, crop rotation, and precision agriculture can enhance production (**c**); microbiome vertical and horizontal transmission (from parent plants to seeds and microbial acquisition from the environment, respectively) can be exploited to protect future crops (**d**).

Below, we explore practical strategies to actively manipulate microbial communities and improve crop resilience. In this section, we discuss the use of additive approaches, passaging, crop management, and **seed microbiomes** for improved crop health (Fig. 3).

### Additive approaches

#### Synthetic communities

When considered alongside inputs that promote selective microbial enrichment or direct addition of beneficial microbes, **synthetic microbial communities** (**SynComs**) serve as a bridge between reductionist microbiome research and real-world applications in sustainable agriculture. SynComs involve the deconstruction and reassembly of target members of microbial consortia based on knowledge acquired from culture-dependent and -independent approaches. The average numbers of microorganisms in these groups range from 2 to 5 members but can be much larger in some cases (39). Considerations for the use of SynComs include methods for community construction and preservation (40), timing of SynCom application (41), and inoculation frequency (42), among other factors.

Armanhi et al. (43) pioneered a community-based culture collection approach to isolate naturally abundant, yet previously understudied, microbial taxa from the sugarcane microbiome (43). The community-based culture approach focused on core microbiota with an abundance-based design (as opposed to trait- or taxonomy-driven screens) which led to successful colonization of plant organs, displacement of native microbiota, and significant enhancement of sugarcane plant biomass (43). There is evidence that specific SynComs can enrich non-member beneficial taxa such as *Actinobacteria* under defined conditions, suggesting that microbial synergy can play a key role in driving plant and microbiome outcomes (44). Though less frequently studied, fungal members of SynComs should not be neglected, as exemplified by the identification of the mycorrhizal species, *Pteridiospora spinosispora*, as a potential candidate for mitigating apple replant disease (45). Applying lessons from SynComs in plant and other systems will enhance our ability to engineer microbial consortia that harmonize with native microbiomes (46).

#### Prebiotics

**Prebiotics** have traditionally been considered in the context of human gut health (47). However, the principles of translational microbiomes suggest that the framework of eubiotic gut microbiomes can also be effectively applied to agricultural systems, particularly regarding healthy soil and plant microbiomes. In plant systems, the term “prebiotics” pertains to a type of soil biostimulants. They are defined as substrates that are selectively utilized by beneficial microbial communities in the soil or phytobiomes, thus enhancing microbial function and ultimately, promoting improved plant health and crop productivity (48). Prebiotic **soil amendment** may more dependably support positive plant health outcomes than strategies that focus on single strain additions, particularly in complex or stressed soil environments (49).

Within the rhizosphere microbiome, nitrogen-fixing rhizobia and arbuscular mycorrhizal fungi represent two of the most extensively studied examples of beneficial plant-associated microorganisms. The application of prebiotics, such as chitin soil amendments, has augmented these beneficial microbiota (50, 51). Other organic soil amendments, like the addition of compost (52), sewage sludge (53), seaweed extract (54), or animal manure (55), are traditional prebiotic practices in agriculture. These materials are considered synbiotic, as they also function as probiotics, a concept discussed in further detail later in this text. These amendments significantly increase microbial biomass and metabolic activity by providing essential nutrients primarily in the form of carbon sources (56). Such amendments have also been demonstrated to enhance the extracellular enzyme activities of soil microbial communities compared to mineral-only fertilization. For instance, Luo et al. (57) demonstrated increased enzyme activities related to carbon, nitrogen, and phosphorus acquisition, alongside increased activity of oxidative decomposition enzymes, highlighting clear biomarkers of microbial metabolic health and robust soil nutrient dynamics. The addition of humic acid is known to improve physical characteristics of soil by improving water-holding capacity and chemical characteristics by bridging negatively charged soil particles to organic anions, forming beneficial soil aggregates (57–60). Humic acid has been shown to boost key soil health indicators and nutrient availability, pH buffering capacity, plant growth, and secondary metabolite production, effects that are underpinned by the upregulation of genes involved in nutrient metabolism and stress response (61). Application of moderate levels of humic acid exerts a prebiotic effect, which may specifically promote bacterial **richness** and suppress pathogenic fungal populations such as certain members of the Ascomycota, and can also favor beneficial saprotrophic and mycorrhizal fungi commonly found among the Basidiomycota and *Mortierellomycota* (62). Recent advances in prebiotic products containing plant extracts, organic acids, trace minerals (e.g., manganese, zinc), and microbial signaling compounds have demonstrated significant enrichment of beneficial microbial taxa such as *Bacillaceae*, *Rhizobiaceae*, and *Pseudomonadaceae* (63–65) and biostimulation of rhizosphere-associated fungi such as *Mortierella* and *Conocybe* (63). Concurrently, these specific enrichments led to the enhancement of plant nutrient cycling capacity, notably through increased enzymatic activity of β-glucosidase alkaline phosphatase (63).

The concept of prebiotics could be expanded to include non-carbon nutrients such as phosphorus-containing materials. For instance, **biochar** serves as carrier material for phosphate-solubilizing bacteria, enhancing its colonizing efficiency, viability, and demonstrated support for tomato plant (*Solanum lycopersicum*) nutrient acquisition (66). Similarly, calcium phosphate (rock phosphate) acts as a selective substrate, enriching soils with microbes equipped to solubilize phosphorus through organic acid secretion systems. This selective enrichment has been demonstrated in the soils of chickpea (*Cicer arietinum*), lentil (*Lens culinaris*), common bean, and maize systems (56, 66, 67). Although these phosphorus substrates may not strictly meet the conventional prebiotic definition, their ability to induce functional prebiotic effects warrants consideration within this translational microbiome framework. Specifically, these substrates can promote beneficial microbial populations through selective pressures that favor balanced nutrient cycling. Phosphate-solubilizing bacteria mobilize inorganic phosphorus and thereby enhance nutrient uptake, which under certain conditions may also compete temporarily with plants for available phosphorus (68). This dynamic interplay between microbial growth and nutrient release highlights the context-dependent nature of prebiotic amendments.

The integration of these prebiotic strategies into agricultural microbiome management aligns with a broader translational microbiome approach, fostering resilience, sustainability, and productivity across diverse agricultural systems. This approach mirrors established models in human gut microbiology, emphasizing targeted nutritional interventions to selectively enhance beneficial microbial consortia, thereby optimizing health outcomes of agricultural hosts and systems. However, the diversity of organisms, environments, and systems makes the design and application of prebiotics in authentic agricultural production challenging, and we describe some of these challenges in the Outlook section.

#### Probiotics

Since the discovery and medicinal application of antibiotics, there has been an interest in applying a contrasting approach—the addition or supplementation of beneficial microbes supporting host health. Werner Kollath coined the term “probiotics” in the 1950s, defined as “active substances that are essential for a healthy development of life” (69). Today, the concept is widely understood as live microorganisms that confer benefit to a host or system when administered at appropriate amounts (70–72). Probiotics, together with the sister concepts of prebiotics, synbiotics (a combination of pre- and probiotics), and postbiotics (microbial metabolites conferring benefits to the host or system), are additive approaches that enrich the utility of microbial communities for translational agricultural benefits (39, 48, 73, 74). In agriculture, the terms “biostimulants” and “biopesticides” are commonly used to refer to the above concepts. Some examples of successful probiotic use in agricultural plants include the application of *Sphingomonas melonis* ZJ26 to seeds to confer resistance against the pathogen *Burkholderia plantarii* (75, 76) and the application of diazotrophic bacteria for successful nitrogen fixation in Brazilian soybean plantations to either partially or even completely replace chemical N fertilizers (75, 77–79).

The supplementation of hosts or cultivation environments with functional microbial communities presents several challenges. First, recurrent supplementation of the probiotic may be necessary. This is because the persistence of strains in a host or environment is still unpredictable due to the complexity of the ecosystem (80). Second, existing probiotics are often limited to bacteria, ignoring important ecological interactions occurring *in situ*. For example, most agriculturally relevant hosts and cultivation environments also contain an abundance of archaea, micro-eukaryotes, and viruses. Third, it is important to note that probiotics or SynComs can harbor genes for antibiotic resistance and other deleterious phenotypes (81). Finally, the standards for evaluating probiotic effectiveness remain limited. A recent study demonstrated that commercially available honeybee (*Apis melifera*) probiotics may even harm their hosts by making them more susceptible to *Nosema apis* infection (82). In the USA, regulations primarily concern biopesticide registration under the Environmental Protection Agency Federal Insecticide, Fungicide, and Rodenticide Act (https://www.epa.gov/pesticide-registration/biopesticide-registration).

#### Antagonists and predators

Notably, there are different ways beneficial microbes confer benefits to hosts or systems. They may supplement hosts with nutrients and growth factors, but they may also inhibit detrimental microbes. Thus, the study and application of microbial predators and **biocontrol** agents is translationally important. For example, bacteriophages are important predators in agricultural systems. Phages have a high level of specificity in their host bacteria, and thus phage applications can effectively reduce target bacterial populations (83). This is especially true if the phages have a predominantly **lytic life cycle** and are applied as diverse communities to reduce the likelihood of the target bacteria developing anti-phage immunity. Another attractive feature of bacteriophages for applications in crop production is that the US Food and Drug Administration considers them as Generally Recognized as Safe (GRAS) substances. Some examples of successful phage applications in crop production include φPD10.3 and φPD23.1 to reduce potato soft rot (84), a cocktail of four phages (*Sano*, *Salvo*, *Prado*, and *Paz*) to reduce Pierce’s Disease on grapevines (85), and a cocktail of 42 phages to reduce bacterial blotch pathogens in white button mushrooms (84–87). Importantly, the emerging research of jumbo phages—phages with genomes >200 kb—has highlighted their potential for phage therapy applications (88). All experimentally isolated jumbo phages are lytic and have effective strategies for avoiding host immunity, drastically slowing the emergence of host resistance. For example, recently, jumbo phages have been shown to generate vesicles and proteinaceous nuclei, shielding their genomes from host methylation and CRISPR/Cas systems (89). There are some companies that are commercializing phages for translational applications, such as Ecophage and Omnilytics.

### Microbiome passaging

In addition to additive interventions, microbial community composition and function can also be shaped through selective pressures over time, as exemplified by microbiome passaging and suppressive soil systems. Microbiome passaging refers to repeatedly subjecting a microbial community to the same environment and associated selective pressures, typically with the goal of enhancing a microbiome-mediated phenotype, such as increasing plant salt tolerance or disease suppression. Depending on the experimental context, approaches that fall under this umbrella have been referred to as “artificial selection” (90, 91), “host-mediated microbiome engineering” (92, 93), or “microbiome breeding” (94).

Microbiome passaging approaches have been applied to both non-host- and host-associated environments. Non-host environments include enhancing pollutant degradation or starch breakdown in laboratory media (95, 96). More recently, Faller et al. (97) used artificial selection to improve phosphate solubilization *in vitro*, a phenotype that was maintained when the community was inoculated into a hydroponic cultivation system with *Chrysanthemum indicum* (97). This result highlights the translational potential of this approach from lab to plant systems. The direct selection of passaged microbial communities within plant-host environments, where the host phenotype is being selected, has also been successfully employed in multiple systems. One of the pioneer studies is by Jochum et al. (92), where rhizosphere communities from drought-resilient wheat plants were selected over six cycles, ultimately achieving a 5-day delay in drought symptom onset (92). Similarly, Mueller et al. (98) selected rhizosphere microbiomes for conferring salt tolerance in *Brachypodium distachyon* under either sodium or aluminum salt stress (98). After nine rounds, specificity emerged: microbiomes selected under sodium stress conferred tolerance only to sodium stress, while those selected under aluminum stress equally ameliorated sodium and aluminum salt stresses. The selected microbiomes enhanced seed production by 55% to 205% as compared to the unselected controls. Studies by Anand et al. (99) and Dubey et al. (100) reinforced the effectiveness of microbiome passaging under salinity stress in *Vigna radiata* (99, 100). Successive microbiome passaging improved plant growth and reduced stress marker accumulation, particularly when salt stress was increased after every other passage. In a recent study, Styer et al. (101) inoculated rice with wild microbial communities and challenged the plants with drought (101). Iteratively selecting microbiomes from the least drought stressed plants across multiple generations, derived simplified microbiomes that enhanced both the growth and drought tolerance of rice.

Passaging has also been extended to less conventional outcomes such as herbivore suppression, disease suppression, and mushroom production. Enders et al. (102) applied the passaging approach for soil microbiome while selecting for high and low insect performance (number of aphids and caterpillar weight) (102). The plant-guided microbiome selection (high vs low selection line) produced temporary effects on aphid populations, whereas caterpillar growth was unaffected. This study highlights both the potential and challenges for the long-term stability of engineered microbial communities. In tomatoes, phyllosphere microbial communities were successively passaged under pathogen pressure (*Pseudomonas syringae* pv. *tomato*; bacterial speck) to enrich microbes associated with low disease severity (103). In mushroom cultivation, O’Connor et al. (104) and Vieira et al. (105) showed that the passaging of casing led to earlier fruiting body formation and faster substrate colonization with a significantly higher mushroom yield in the first flush although the total mushroom yield was not impacted (104, 105). O’Connor et al. (104) further demonstrated that along with stimulating early pinning, passaged casing also altered disease dynamics: passaged casing had a suppressive effect on blotch disease (*Pseudomonas tolaasii*) and a conducive effect on green mold disease (*Trichoderma aggressivum* f. *aggressivum*) (104). Although this observation has not been replicated, these findings highlight potential off-target outcomes that may emerge during microbiome manipulation.

For successful translation, the stability and heritability of selected traits are crucial. Jacquiod et al. (106) addressed this by selecting rhizosphere microbiota from *Brachypodium distachyon* plants for high or low leaf greenness over 10 generations (106). The authors observed a two-phase process: an initial transitory phase with community instability and no heritability of the selected property, followed by a stabilization phase where the microbiota structure stabilized and the selected property became heritable. Notably, when the 10th-generation selected microbiota was inoculated on four different *Poaceae* plant species cultivated on two different soils, it was observed that the effect of microbes was dependent on plant species but not on the soil type, underscoring host specificity in microbiome function (107).

To robustly evaluate the outcomes of microbial passaging and selection, studies use multi-line experimental designs including high-selection lines (microbial communities ranking highest for the phenotype of interest), low selection lines (those ranking poorly), random selection lines (no phenotype-based selection), and null control (no inoculation or inoculating with sterilized community). However, in some cases, a low-selection line can be problematic. For example, Jochum et al. (92) reported that selection based on a low-scoring plant phenotype (poor growth under drought conditions) caused the accumulation of pathogens in the soil, a phenotype that was not targeted for selection (92). In other studies, null or sterile inoculated controls failed to serve the purpose. For instance, in studies by Ehau-Taumaunu and Hockett (103) and Styer et al. (101), microbial migration or dispersal from surrounding environments limited their usefulness as baselines (101, 103). Thus, establishing appropriate controls can be difficult. Various challenges faced during these microbial community selection experiments have also been highlighted by Arias-Sánchez et al. (108) that include but are not limited to (i) unstable ecological dynamics, (ii) short selection rounds, or (iii) emerging mutants (108). These challenges highlight the need for careful experimental designs and careful interpretation of selection outcomes. Some studies, such as Styer et al. (101), also introduced an initial enrichment generation, a pre-selection cycle without any stress treatment, to first enrich microbiota associated with healthy hosts (101). This step ensures a more stable and potentially beneficial starting community before applying selective pressures in subsequent cycles.

Translating microbiome passaging approaches to production agriculture will require adapting these controlled, iterative selection systems to the complexities of open-field conditions. While there is precise control in lab and growth chamber conditions, scaling this approach to commercial systems would likely involve initial microbiome selection cycles in controlled settings and then testing evolved microbial consortia in field conditions. The applications could be integrated with existing grower practices such as seed coating, foliar sprays, or compost inoculation. However, successful translation will depend on the stability, formulation, and regulatory approval of these selected consortia, as well as understanding how environmental heterogeneity, crop rotation, and agrochemical inputs influence their persistence and performance (L. Kaur, T. H. Bell, J. Sadeghi, and K. L. Hockett, unpublished data).

### Agricultural management

The principles of microbial selection observed in passaging experiments are also at play in real-world agricultural systems. Management practices, such as tillage or fertilizer use, can exert selective pressures that alter microbial community structure and function akin to what is observed during passaging. Tillage intensity (109), cropping history (110), soil amendments (111), irrigation (112) type, crop genotype (113), and chemical and organic inputs (111, 114) all contribute to microbiome variability (109–114). However, given the multifactorial nature of research in agroecosystems, it has been challenging to determine how to use different management practices to build microbiomes that support crop health. For example, one of the most widely applied changes in management to enhance plant health is a reduction in tillage intensity. While this reduction has been demonstrated to improve soil health and increase soil microbial biomass, their effect on plant performance has not been consistent across different crops (109, 115).

Similarly, soil amendments, like organic fertilizers, have been evaluated for their effect on rhizosphere and soil microbial communities. Organic fertilizers influence microbial community composition in the soil; however, this does not always translate to higher plant biomass or yield. For example, an experiment comparing animal-based fertilizer to plant-based fertilizer found that while each led to a different soil microbiome profile, there were no differences in plant biomass by the end of the experiment (116). This may be due to the significant impact of other factors on plant performance. This was evident in a study by Bossolani and colleagues, which examined the effect of plant organic amendments with different C/N ratios on microbial community composition and soil properties (117). Here, although each amendment led to different microbial community profiles, there was no significant driver of increased plant biomass (117).

The use of animal derived fertilizers like manure garnered scrutiny due to concerns on the risk of increased antimicrobial resistance (AMR) genes entering our cropping systems. AMR predates the discovery of antibiotics (118). However, the emergence of multidrug-resistant pathogenic bacteria has prompted more exhaustive analyses of the sources and movement of AMR genes (119). Factors such as wastewater management, irrigation water, run-off from animal production environments, as well as both wild and domestic animals, can be sources of AMR (120–124). The use of manure as fertilizer is one of the reservoirs that can be more readily managed. Adequate composting has been shown to significantly reduce the abundance of AMR bacteria and genes within manure (125, 126). Even after composting, the repeated application of manure has been shown to increase AMR in soil (121, 127). Some studies have found that although animal-derived composted material can increase AMR gene abundance in soil, this increase may be short-lived and not significant in the final product (128, 129). In contrast, AMR microbes and genes have been found in consumer produce purchased from different vendors, while controlled studies have found an increase in AMR genes on produce grown on animal-derived compost material (130–132). These contrasting results point to multiple reservoirs influencing the AMR gene pool of the final product. Using metagenomic approaches to track the movement of these AMR genes across reservoirs can aid in identifying where and when they proliferate to better inform management decisions.

Management practices that increase microbial diversity have been broadly characterized as beneficial across different agricultural systems. This diversity can be supported through various practices, like the addition of organic amendments, increasing crop diversity, and reducing chemical inputs. Although these practices can increase phytobiome diversity, it is also important to highlight that higher diversity is not always supportive of better plant performance. For example, a dilution experiment in barley, where alpha diversity was reduced in soil, found that less diverse microbiomes led to higher plant biomass (133). This experiment was terminated 3 weeks after sowing. Hence, these conclusions are only representative of short-term effects of different microbial diversities on plant biomass. While supporting biodiversity is of interest in its own right, using microbial diversity as a measure of positive impacts in agricultural systems should be critically evaluated, especially across experiments with different timeframes.

It is clear that plant genotype is one of the most significant sources of microbiome selection. This has been demonstrated in various crop plants, including soybean (134), rice (135), wheat (136), cotton (137), and sugarcane (138), where different genotypes of the same species generated different microbiome assemblies (134–138). Moreover, these microbiome differences have been associated with changes in plant yield for both soybean and rice (136, 139). Interestingly, a study by Xiong et al. (135) highlights the interplay of plant genotype, fertilization regime, and microbial community assembly on the resulting yield of rice plants (135). As more of this research is done, we may be able to make recommendations for cultivar selection based on the available microbial background and the microbial recruitment of specific genotypes. This underscores the importance of continuing to conduct research that disentangles how other management practices influence microbial communities. With this in mind, there has been a growing interest in microbiome-conscious plant breeding (139, 140).

Some companies are at the forefront of using microbiome insights to drive management decisions. Trace Genomics, for example, combines microbial community data and soil properties to help farmers in making decisions about seed selection, targeted nutrient, and pest management. Another company that has embraced and harnessed the complexity of microbial communities for enhanced crop management is Jord BioScience. This company aims to improve the performance of both conventional and biological inputs through custom microbial consortia that interact synergistically with these inputs. Both companies have reported significant increases in yield, which paints an optimistic picture for further applications of microbiome-conscious management and targeted solutions.

### Seed and propagule microbiomes

Although agricultural management can modulate microbiomes at multiple stages of plant development, the seed microbiome represents a unique and foundational reservoir that is increasingly recognized for its role in plant health and transgenerational microbial transfer. The seed microbiome encompasses a diverse community of microorganisms, including bacteria, fungi, archaea, and viruses, which colonize seeds either **epiphytically** or **endophytically**, playing critical roles in plant health from germination to maturity (141, 142). These microorganisms are not exclusively passive inhabitants but actively influence various aspects of seed germination, plant growth, and overall plant health (143). Translationally, seed associated microbes offer practical routes to reduce chemical inputs, via pathogen suppression and improved early establishment (e.g., phytohormone production, ACC-deaminase activity, nutrient mobilization), linking directly to measurable gains in plant health and productivity (144, 145).

Functionally, seed microbiomes serve as microbial reservoirs that impact subsequent plant generations (146). They significantly improve germination rates, seedling vigor, and establishment success while modulating key plant functional traits (147, 148). These communities may enhance nutrient availability and uptake while conferring tolerance to both biotic and abiotic stresses (145, 149). Specific microbial taxa may also directly contribute to disease resistance and growth promotion through multiple mechanisms including induced systemic resistance and the production of antimicrobial compounds (145, 150). Endophytic microorganisms particularly influence agricultural outcomes by improving yields and crop quality (151), while overall microbiome composition affects critical plant characteristics from leaf longevity to biomass allocation (152).

Plant microbiomes assemble through both vertical transmission from the parent plant through seeds and horizontal transmission of microbes from the environment to the plant (153). Microbial colonization patterns are shaped by complex interactions between floral pathways, seed structures, and environmental conditions during development (153, 154). While environmental factors dominate microbiome composition, the plant genotype also exerts significant influence, enabling targeted breeding approaches (155). Agricultural practices such as the application of fertilizers dramatically alter seed microbial communities, highlighting opportunities for management-based optimization (156). This understanding has informed strategies to engineer beneficial microbiomes through both breeding programs and agricultural interventions (144, 156, 157).

In disease management, seed microbiomes provide natural protection against pathogens through **competitive exclusion** and induced resistance (145, 158). Native endophytes contribute substantially to plant innate immunity (150), with specific taxa like *Paenibacillus* showing demonstrated efficacy against bacterial wilt in tobacco (150). Practical applications include seed coatings with *Trichoderma* or *Bacillus* species that enhance plant health while improving rhizosphere conditions (159, 160). These approaches align with sustainable agriculture goals by reducing chemical pesticide use while maintaining productivity (145, 161). However, it is important to consider that seed microbiomes themselves may harbor plant pathogens or other detrimental organisms. This makes detection of plant pathogens in seed microbiomes essential, allowing us to target those organisms using traditional methods and beneficial components of the seed microbiomes, as described above in the Intervention Strategies section.

This conceptual framework can be extended beyond seeds to other reproductive organs involved in vegetative propagation. Beyond seeds, clonally propagated storage organs such as tubers, bulbs, and corms function as vertically inherited microbial reservoirs with seed-like consequences for establishment, health, and quality. Seed tubers carry distinct, compartmentalized endophytic communities that reflect production field and cultivar and remain detectable after storage; when planted, field-of-origin effects persist in daughter tissues, consistent with seed-tuber imprinting across generations (162, 163). Seed-tuber lots with contrasting disease phenotypes also differ in microbial composition (164), suggesting microbiome-informed screening of planting material. In bulb crops, storage onions harbor structured bacterial and viral communities linked to bulb-rot status, while garlic and other *Allium* species carry structured endophytic bacterial communities with documented roles in growth promotion and biocontrol (165). In saffron, endophytic communities are linked to variation in crocin and related metabolites, and corm-rot outbreaks are characterized by dysbiosis with *Fusarium* dominance, conditions that jeopardize yield and quality (163, 166). Collectively, these studies extend the seed-microbiome concept to vegetative propagules and motivate phytosanitary profiling and targeted enrichment of beneficials across reproductive/storage organs and, where relevant, integration with pathogen diagnostics such as those reported for saffron corm rot (167). Modulation strategies for seed microbiomes include direct inoculation, floral treatments, and selective breeding (147, 157, 159). Microbial consortia and plant-growth-promoting rhizobacteria inoculants enhance multiple plant traits simultaneously (168, 169), with seed coatings delivering targeted benefits to both plants and soil (159). These interventions translate into measurable agricultural improvements including yield increases and enhanced stress resilience (147, 149). Particularly under climate change pressures, tailored microbiomes offer solutions for maintaining productivity in challenging environments (153).

Seed microbiomes also contribute to crop **terroir** by influencing phytochemistry and product qualities (170). Microbial modifications of host metabolism affect nutritional content and sensory characteristics in economically important crops like coffee and wine (170). Agricultural practices that shape seed microbiomes consequently impact end-product attributes, creating opportunities for quality optimization through microbiome management (145, 170).

Metagenomic profiling of seed and seedling microbiomes recovers functional genes tied to phytohormone metabolism (e.g., *ipdC*, *iaaH*, *acdS*) and nutrient cycling (e.g., pqq-mediated phosphate solubilization), functions that map to germination and early seedling physiology; when integrated with metabolomics, such data sets can be linked to host metabolite profiles and physiological traits (171, 172). High-throughput sequencing and bioinformatics enable comprehensive community analysis (157, 160), while multi-omics approaches provide a systems-level understanding of plant-microbe interactions (173). These tools facilitate targeted manipulation of microbiomes for crop improvement (174), with emerging techniques like epigenetics offering new research avenues (152).

Significant knowledge gaps remain regarding transmission mechanisms and functional specificity of seed microbes (146, 153). Practical challenges include environmental variability in microbial performance, the shelf life of microbial seed treatments, and scaling hurdles for commercial applications (175). Future directions should integrate genomics and synthetic biology with precision agriculture (175), while investigating epigenetic influences on seed quality (173). Translational success will require collaborative efforts across sectors to bridge research and implementation, ultimately realizing the potential of seed microbiomes for sustainable agriculture (175).

## OUTLOOK

The American Phytopathological Society “Phytobiomes Roadmap” indicates that the emergent properties of plant microbiomes are greater than the sum of their parts (176). This apparent synergy may be the product of not yet understanding—or, even, having all the necessary tools to understand—the interactions within phytobiomes eliciting observed phenotypes. In other words, we have a long way to go before we can wave our “tricorders” over a plant sample or field, à la *Star Trek*, and know exactly what amendments or microbes are needed. In the sections above, we describe efforts to deploy microbiome-informed methodologies aimed at improving crop production. Some of these approaches are more mature and broadly used (e.g., phage therapy and agricultural management), while others are more novel and face challenges to translation due to their complexity, novelty, and immature regulatory environment (e.g., SynComs, passaging, and precision agriculture). Additionally, these techniques are just beginning to scratch the surface of the exploitable complexity of microbial communities in agricultural systems. We believe there are a series of core objectives which must be met to most effectively apply microbiomes translationally.

First, due to the complexity and variability of agricultural microbiomes, precision agriculture should be a priority. This concept is analogous to the movement toward personalized medicine. The literature is replete with data demonstrating the impact of environmental variability on the success of biological control treatments. For microbiome-informed treatments to be successful, we will need to be able to provide “personalized treatments” for crops, treating individual fields, parts of fields, and, potentially, individual plants. Deploying microbiomes as diagnostic tools using ‘omics techniques, such as precisely identifying pathogen strains or stress-related community shifts, and screening for fine-scale spatial variations, such as using near-infrared light to inform fungicide application, are examples (177). Crandall et al. (178) propose including “spectranomics” for characterizing foliar functional traits via differential, remotely captured hyperspectral images (178). Also, a variety of crop stresses leading to dysbiosis are detectable using these technologies. As ‘omics techniques and advanced farm equipment become cheaper and more widely available to growers, the use of microbiome-aware precision agriculture will likely grow. These efforts to model and deploy microbiome interventions would also greatly benefit from improved metadata standards.

In addition to developing “personalized treatments” for plants, there are long-standing questions in biological control research that, if answered, would significantly contribute to the advancement of translational microbiomes for crops. One recalcitrant issue is the lack of correlation between *in vitro* and *in vivo* or *in situ* evaluations of products or practices (179). While translational microbiome studies still rarely extend past *in vitro* experiments, only *in vivo* and *in situ* trials can establish whether these treatments are impactful despite the background of batch effects and biological and ecological complexity. Identifying principles that translate to management at scale across the variation in fields, farms, and regions will require significant investment. An approach that avoids the pitfalls of translating *in vitro* studies to crop systems is passaging, which is always conducted *in vivo*. However, passaging approaches are still typically conducted under highly controlled conditions, and the validation of these approaches on farms is needed. There are efforts to apply passaging in production systems, such as in white button mushroom production.

Third, microbiome products for growers must be regulated and commercialized. This process will set standards for product safety, effectiveness, and reproducibility. Several key steps toward this objective include obtaining patents, regulating the design and use of microbial consortia, and regulating crop management practices and extension efforts. However, there are no regulatory protocols for evaluating microbiome-based products (180). A framework for evaluating safety for microbial products was established in 1996 (181) which could be revisited for microbiome-based products. In the same vein, microbiome products and commercialized methods should take advantage of existing agricultural companies for marketing and distribution. This should lower the activation energy required to bring these techniques to the market. However, recommendations for on-farm management practices that promote eubiotic microbiomes may be the most cost-effective option for growers and would require less regulation.

Finally, perhaps the most important objective is to develop relationships and trust with stakeholders. Grower acceptance is a major hurdle, and extension research remains a major bridge toward grower acceptance. Participatory research (in which growers are co-designers of research) is a successful approach leading to acceptance and adoption (182, 183). With appropriate translation of the technology, participatory research could encourage *in situ* testing of translationally applicable microbiome attributes, benefiting growers, consumers, microbiome companies, and scientists. In the coming years, it will be crucial to not over-promise or over-sell microbiome products, applying research findings conservatively. It is important to note that organic growers are among the most microbiome-aware producers due to their restrictions in using petroleum-based fertilizers, synthetic pesticides, and other synthetic products (184). They continually prioritize soil microbiome research to advance disease management and nutrient cycling in their production systems (184).

We propose that the best tools at scientists’ disposal to address the above objectives are carefully designed, long-term *in situ* experiments. These experiments should be established in locations with different edaphoclimatic conditions to represent the diversity of agricultural systems. By providing both time and diversity of conditions, scientists will be able to better understand the influence of microbiome interventions on crop health. In addition, we would be able to understand the impact of these approaches in the long term. This will inform not only about the effectiveness of an intervention but also its durability, potential off-target effects, and generalizability across different agroecosystems. Here, we have focused on how to enact microbial community change through different tools though it is important to highlight that understanding plant response is an important piece of the puzzle. Determining the primary mediators of the beneficial effects of different interventions will help us in developing more robust solutions. This means that we need to employ a holistic approach that considers environmental conditions, microbiome function, and plant response.
